# Molecular predictors of treatment resistance and recurrence following neoadjuvant therapy in rectal cancer

**DOI:** 10.1186/s12885-025-14958-4

**Published:** 2025-10-22

**Authors:** Fengyuan Huang, M. Chandler McLeod, Regina K. Irwin, Mary Smithson, Min Gao, Karin M. Hardiman, Zechen Chong

**Affiliations:** 1 Department of Biomedical Informatics and Data Science, Heersink School of Medicine, University of Alabama at Birmingham, Birmingham, AL 35294 USA; 2 Department of Genetics, Heersink School of Medicine, University of Alabama at Birmingham, Birmingham, AL 35294 USA; 3 Department of Surgery, Heersink School of Medicine, University of Alabama at Birmingham, Birmingham, AL 35233 USA; 4 Department of Medicine, Division of Cardiovascular Disease, Heersink School of Medicine, University of Alabama at Birmingham, Birmingham, AL 35233 USA; 5 Department of Surgery, Birmingham VA Medical Center, Birmingham, AL 35233 USA

**Keywords:** Neoadjuvant therapy, Rectal cancer, Genomic and transcriptomic characteristics

## Abstract

Neoadjuvant chemoradiotherapy (nCRT) is the standard treatment for locally advanced rectal cancer, but only 20–40% of patients completely respond to this treatment. Pre-treatment predictors of response are understudied. To define the molecular features that are associated with response to nCRT, we compared genomic and transcriptomic data from 318 pre-treatment biopsies of microsatellite stable rectal cancers from our own data and from publicly available data. We found that patients with a complete response have decreased risk of both local recurrence and future metastasis. We identified multiple differences in DNA mutations and transcripts between complete and incomplete responders. Different genes contained significantly co-occurring and mutually exclusive mutations between CR and ICR tumors. In addition, complete responders had lower expression of genes enriched with multiple mechanisms of DNA repair indicating that defective DNA repair may be associated with complete response to nCRT. We developed a logistic regression model adjusted for tumor size, stage, and age to determine the relationship co-occurring mutations and tumor complete response and found that the number of certain co-occurring mutations to be highly predictive of CR. When we assessed for predictors of recurrence in incomplete responder tumors, abnormal cell–cell interaction and increased cancer associated fibroblasts were associated with recurrence. Additionally, gene expression analysis identified a subset of immune hot tumors with worse outcomes and upregulation of immune checkpoint proteins. These data provide additional understanding of the molecular features associated with response to nCRT and the underlying molecular differences in responders and non-responders.

**Supplementary Information:**

The online version contains supplementary material available at 10.1186/s12885-025-14958-4.

## Background

Colorectal cancer is the third leading cause of cancer death in the United States [1]. Rectal cancer constitutes about one-third of colorectal cancer cases but the rate of rectal cancer is increasing, especially among young people [2]. The standard recommendation for Stage 2 and 3 rectal cancers is for patients to undergo treatment with neoadjuvant chemoradiation (5FU and 2 Gy radiation for six weeks) and often chemotherapy (FOLFOX) referred to as total neoadjuvant therapy (TNT), followed by surgical excision using the total mesorectal excision technique [3]. Responses to this neoadjuvant treatment vary substantially [4], with complete response rates between 20 to 40% [5]. Complete responders have reduced risk of local recurrence and metastasis [6, 7] and as demonstrated in a recent multicenter trial, these patients likely do not need to undergo total mesorectal excision [5]. It is worth noting that the correlation between short-term outcomes and long-term survival has some limitations in the context of TNT trials [8], where factors such as patient heterogeneity and treatment variability may confound these associations. The lack of predictors of response remains a significant obstacle to improving treatment outcomes and omitting surgery.

Prior research has assessed clinical predictors of complete response, including increased serum carcinoma-embryonic antigen (CEA), imaging-detected extra-mural venous invasion and tumor size, primary tumor and regional lymph nodes staging (T and N stages) [9]. Clinical predictors of overall worse outcome generally predict worse response to neoadjuvant therapy, but no prior study has identified genomic predictors that are used clinically to predict which patients will be complete responders to neoadjuvant treatment.

As technology advances, studies have begun to assess alterations in DNA, RNA, and individual proteins as well as immune cells as predictors of response [10–14]. Smaller studies of individual or small groups of genetic alterations have yielded mixed results. A recent meta-analysis that assessed the prognostic role of specific genomic mutations (*RAS*, *TP53*, *BRAD*, *PIK3CA*, and *SMAD4*) in predicting complete response found that only *KRAS* mutation was significantly associated with incomplete response [15]. Most of these studies had small sample sizes (< 30 patients) and didn’t integrate RNA and DNA sequencing data [4, 16]. There have now been multiple small studies of circulating DNA assessing its utility as a predictor of complete response which have not found it to have high enough sensitivity to be useful this purpose although it does appear to predict which colon cancer patients will benefit from chemotherapy [17, 18]. The larger dataset from the Cancer Genome Atlas (TCGA) rectal cancer cohort excludes treated tumors and thus, this is unable to address the question of why patients respond differently [19]. A recent study utilizing genomic data on 738 rectal cancer patients provided a summary of genomic data but lacked in-depth integrated analysis assessing for predictors of response [20].

**Fig. 1:**
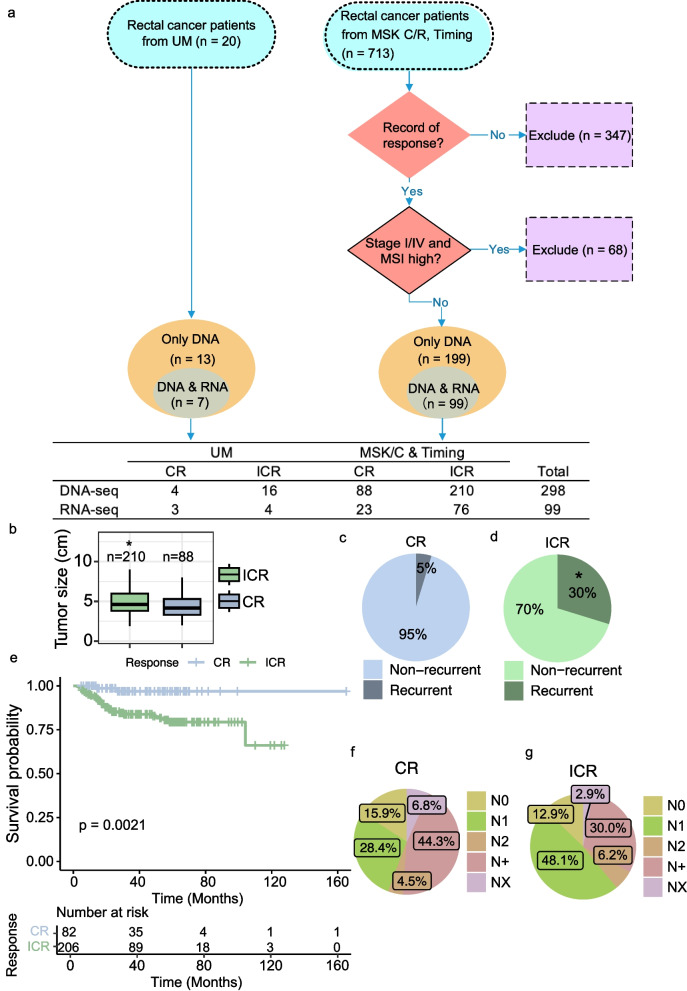
Summary of Data sources and clinical differences in response to neoadjuvant therapy (nCRT). **A** Overview of the datasets used in the study. UM: University of Michigan cohort; TIMING: Timing of Rectal Cancer Response to Chemoradiation cohort; MSK C + R: Memorial Sloan Kettering Research + Clinical cohort. Patient selection is depicted, including exclusion criteria and the counts of patients whose DNA and RNA were sequenced. **B** Tumor Size Distribution by Response to nCRT. Box plots display tumor sizes comparing complete responders (CRs, *n* = 88) to neoadjuvant chemoradiation therapy (nCRT) with incomplete responders (ICRs, *n* = 210). Statistical analysis using the Mann–Whitney U test shows a significant difference (*p* = 0.0368). Sample size for the analysis totaled 298 patients. **C**-**D** Pie charts showing the fractions of recurrent in complete responders (CR, *n* = 88) and incomplete responders (ICR, *n* = 210), respectively. **E** Kaplan–Meier survival curve showing the survival difference between complete responders (CR, *n* = 88) and incomplete responders (ICR, *n* = 210). **F**-**G** Pie charts showing the fractions of N stages in complete responders (CR, *n* = 88) and incomplete responders (ICR, *n* = 210), respectively. An asterisk in panel **C** signifies a statistically significant difference as determined by the Chi-squared test (*p* = 8.007e-07)

Our study combined genomic sequencing data from pre-treatment biopsies of 20 rectal cancer patients from our group with publicly available data from 298 patients. We excluded patients with missing data, stage 1 or 4 disease, and MSI-H tumors (Fig. 1A) [20]. Because our data was collated from multiple studies, it included patients who had a variety of treatments, but all underwent treatment with 5-fluorouracil plus long course radiation as part of their treatment. We performed an in-depth integrated analysis to identify response-associated alterations and features linked to future recurrence. We specifically performed analysis not performed in the recent manuscript by Chatila, et al. [20] using much of the same data. We focused specifically on the role of particular pathway alterations (both DNA and RNA) in treatment response and recurrence, mutation rate in response to treatment in MSS tumors, and the role of interactions between mutations because we hypothesized that interactions between pathways and mutations would potentially predict outcome rather than individual genes.

## Methods

### Patients

For this study, we identified and analyzed available data for patients who had pre-treatment tumor biopsies with subsequent DNA and/or RNA sequencing of their tumor, received chemoradiation, had subsequent surgical resection or clinical followup as part of a trial, and had available data regarding tumor response to treatment. Tumors were from four cohorts: University of Michigan (UM) (20 patients), Timing of Rectal Cancer Response to Chemoradiation (TIMING) (66 patients), and Memorial Sloan Kettering (MSK) Research and Clinical cohorts 232 patients) were included, totaling 318 patients with confirmed diagnosis of rectal adenocarcinoma (Fig. 1a). The UM cohort was collected personally from consented patients by KMH while at that institution. The UM patients underwent treatment with 5FU and 54 Gy radiation over 5.5 weeks (chemoradiation) followed by surgery 8–12 weeks later. The TIMING trial patients got chemoradiation followed by differing numbers of cycles of FOLFOX (5FU, oxaloplatin, and leukovorin) prior to surgical resection. The MSK cohort got chemoradiation either before or after 8 cycles of FOLFOX and patients who appeared to have a clinical complete response on endoscopy and imaging were followed. For our study, we considered patients to have a CR if they underwent surgical resection and had a CR or if they were a persistent clinical CR after 2 years of follow up in the MSK dataset but did not undergo surgical resection. All genomic and associated clinical data for MSK and TIMING were downloaded from the cBioPortal (https://www.cbioportal.org/study/summary?id=rectal_msk_2022). Raw RNA sequencing data from all cohorts were downloaded from GEO under the accession numbers: GSE242786 and GSE209746.

### Whole exome sequencing

For the UM cohort, genomic DNA samples were fragmented to a target size of 300 bp using the Covaris S2 system. The samples were end-repaired, poly A-tailed, and Ligated with custom adapters using the NEBNext DNA Library Prep kit. These adapters had 6 bp barcodes designed with BARCRAWL software and synthesized by Integrated DNA Technologies. After Ligation, they were size selected to 300 bp on a 2% agarose gel, retaining 1-mm gel slices. The samples were isolated from the gel using the Qiagen QIAquick system. Ligation products (10 or 15 μl) were enriched using the Phusion master mix kit and underwent 14 PCR cycles. PCR products were purified using AmpureXP beads. Library quality was checked using the Agilent Bioanalyzer and qPCR. The libraries were captured using the Nimblegen SeqCap EZ V3 Exome Enrichment Kit and sequenced on the Illumina HiSeq 2000 with 100 bp paired-end reads using v3 reagents.

### Somatic mutation detection

For detecting somatic single nucleotide variant (SNV) and insertions/deletions (INDELs), the best practice guidelines of GATK (v4.0.11) [21] (https://software.broadinstitute.org/gatk/best-practices/workflow?id=11146) were followed, and strelka2 (v2.9.10) [22] was employed. Somatic variants with a depth < 30 and Allele Fraction (AF) < 0.1 were excluded. The remaining mutations were annotated using ensembl-vep (v109.3) [23]. The VCF files were converted to MAF format using vcf2maf (v1.6.21) [24]. Analysis focused on mutations in coding regions. A mutation rate (total mutations/length of coding regions) was calculated for all somatic mutations. A propensity score weighting algorithm (PSW) [25] was applied to balance confounding factors. The propensity score was calculated based on the response type (CR and ICR) using a logistic regression. Samples were re-weighted using the Matching Weight scheme (MW) [26]. This process included balanced checking steps that revised the calculation until standardized differences of all covariates were less than 10%, balancing confounding factors between CR and ICR patients. Next, mutation rates between the two balanced groups were compared using a weighted t-test.

### Significantly mutated genes detection

MutSig2CV [27] was applied to identify significantly mutated genes in tumors with complete response and incomplete response to neoadjuvant chemoradiotherapy (nCRT). Significantly mutated genes were mutated beyond random expectations, considering the background mutation rate and mutational processes. Genes with *q*-value < 0.05 in CR tumors, but > 0.05 in ICR tumors were defined as CR-specific, and vice versa for ICR-specific genes. Functional enrichment analysis of SMGs was performed using Enrichr [28]. Significantly enriched Gene Ontology (GO) and Kyoto Encyclopedia of Genes and Genomes (KEGG) pathways were identified with a threshold p-value < 0.05.

The mutated genes with > 5% mutation frequency were selected for comparison between CR and ICR, including recurrent and nonrecurrent ICR tumors. Co-occurring and mutually exclusive mutation pairs were analyzed in CR and ICR tumors separately using the maftools(v3.15) [29] package. Interactions of top 25 mutated genes were visualized using the ggplot2 package. Networks and interactions of these mutations were explored in the STRING database [30] and illustrated via Cytoscape (v3.10.0) [31].

### RNA sequencing

From our cohort, RNA was isolated from fresh frozen pre-treatment biopsy samples using the Allprep kit (Quiagen). Total RNAs were used to generate mRNA sequencing Libraries and sequenced on the Illumina HiSeq 2000 platform. The TIMING and MSK samples were treated similarly [20].

### Transcriptomic analyses

Raw FASTQ files were assessed using FastQC (v0.11.9). Single-end RNA-seq reads were aligned to the human genome (GRCh38) with STAR software (v2.6.1) [32]. Gene expression levels were calculated from BAM files using HTSeq (v0.11.2) [33]. Raw read counts were normalized, and genes with a p-value ≤ 0.05 were identified as differential expression genes (DEGs) using the Deseq2 package (v3.14) [34]. Functional enrichment of DEGs was performed through Enrichr [28]. Significant GO terms and KEGG pathways were identified with p-value < 0.05. Furthermore, Gene set enrichment analysis (GSEA) [35] was conducted to determine gene sets that were significantly different between response and non-response groups. The H (h.all.v6.2.symbols.gmt) sub-collection from the Molecular Signatures Database (http://software.broadinstitute.org/gsea/msigdb/index.jsp) served as the reference for GSEA, with a normalized enrichment score (NES) and false discovery rate (FDR) denoting statistical relevance.

For the validation of subset ICR tumors using TCGA rectal tumors (READ), the gene expression data processed using the second TCGA analysis pipeline (RNAseqV2) were collected. Only tumors in stage II and III with consensus molecular subtypes (CMS) subtypes and microsatellite stable, were included. The hierarchical clustering method was used to identify cluster 4-like tumors based on the DEGs between cluster 4 and other clusters.

### Immune cell infiltration analyses

Based on gene expression, abundances of infiltrating immune cells were estimated using TIMER2.0 [36] which includes TIMER, CINERSORTx [37], quanTIseq [38], xCell [39], MCP-counter [40] and EPIC [41] algorithms. Significant differences in immune cells between tumor groups were defined at the cutoff of *p*-value = 0.05 using the Mann Whitney U test.

### Survival analyses

The “Survival” package (v3.3–1) was used to build a standard survival object. To evaluate gene expression on survival, Kaplan–Meier survival curves were generated. Patients were stratified into high and low expression groups based on mean expression levels. Survival curves for these groups were compared using proportional hazards assumptions, and the log-rank test was used to evaluate significant differences in survival durations (*p*-value < 0.05).

### Logistic regression model for complete response

To identify potential predictors of tumor response, a logistic regression model [42] was fit to clinical and genetic data. First, bivariate comparisons using t-tests, Wilcoxon rank sum and Fischer’s exact test were made, as appropriate, between tumor response and patient demographics as well as binary indicators for co-occurring mutations. Demographic and clinical variables with *p* < 0.2 were selected for inclusion while co-occurring mutations associated with greater CR at *p* < *0.2* were incorporated into the model as a count of the number of co-occurring mutations per tumor. The logistic regression model for predictors of complete response was then fit including tumor size, tumor stage, patient age, and number of co-occurring mutations with all except stage utilized as continuous variables. Model performance metrics including sensitivity, specificity, positive prediction value (PPV), and negative prediction value (NPV) were evaluated on the training data using the R package pROC [43].

### Statistical analysis

Statistical analyses were conducted using R Studio (v1.2.1335) and R (v3.6.1). Specific R packages and methods used in each step are described in the preceding sections.

## Results

This study analyzed stage 2 and 3 rectal cancer patients who received chemoradiation post-biopsy from three cohorts: 20 from University of Michigan (UM), and 298 from the Memorial Sloan Kettering (MSK) groups and Timing of Rectal Cancer Response to Chemoradiation (TIMING) cohort. Of the original 733 patients across the samples, we excluded 347 patients who lack of record of response and 68 microsatellite instability-high tumors or tumors in stages I/IV. Subsequently, 318 patient tumors with available DNA or RNA sequencing data were used for our genomic analyses (Fig. 1A). Those showing pathological or durable (at least 2 years) clinical complete response were categorized as complete responders (CRs), while the rest were classified as incomplete responders (ICRs). We performed a comprehensive analysis of the data available for these complete responders and incomplete responders. Which samples were used for which analysis was dependent upon available data and is described in detail (Supplementary Fig. 1 A). Significant differences in the available clinical variables and outcomes were identified between the CR (n = 88) and ICR groups (n = 210) (Supplementary Fig. 1B). Notably, ICR patients exhibited larger tumor sizes (Mann Whitney U test, *p*-value < 0.05, Fig. 1B), higher recurrence rates (Chi-square test, *p*-value < 0.05, Fig. 1C-D) and decreased overall survival (Log-rank test, *p*-value = 0.002, Fig. 1E; CR median follow-up time: 33.8 months, ICR median follow-up time: 43.6 months). Furthermore, 84.2% of ICR patients were clinical lymph nodes positive (cN1, cN2, cN +), compared to 77.3% of CRs (Fig. 1F-G). These results align with earlier research linking complete response and reduced recurrence [44].

### Genomic characteristics of response to nCRT

Prior study has indicated that complete response to nCRT is associated with a high tumor mutation burden (TMB) in rectal cancer [45] but potential patient related confounding factors were not accounted for such as BMI, gender, and age. To do so, we applied the propensity score weighting algorithm to the TMB comparison (Supplementary Fig. 2 A). After this adjustment, the statistical comparison of TMB is not significant but CR tumors exhibited a mutation rate twice as high as that of ICR tumors (Supplementary Fig. 2B, p = 0.46). Important to note, this increased mutation rate in CR tumors was not influenced by the presence of microsatellite instability (MSI) in tumors as only MSS tumors were included in our study. This finding implies that TMB may be related to confounding factors accounted for in our analysis.

We also aimed to identify the relevant genetic features associated with response. We first focused on the frequently mutated genes and compared their mutation frequencies between CR and ICR tumors. While no significant differences were identified, commonly mutated colorectal cancer-related genes *TP53*, *TTN, MUC16*, *APC* and *KRAS* were found in a high percentage of all tumors (Supplementary Fig. 2C-D). However, genes *APC, KRAS, FBXW7* and *PIK3CA* displayed higher mutation frequencies in ICR tumors (Supplementary Fig. 2D).

**Fig. 2:**
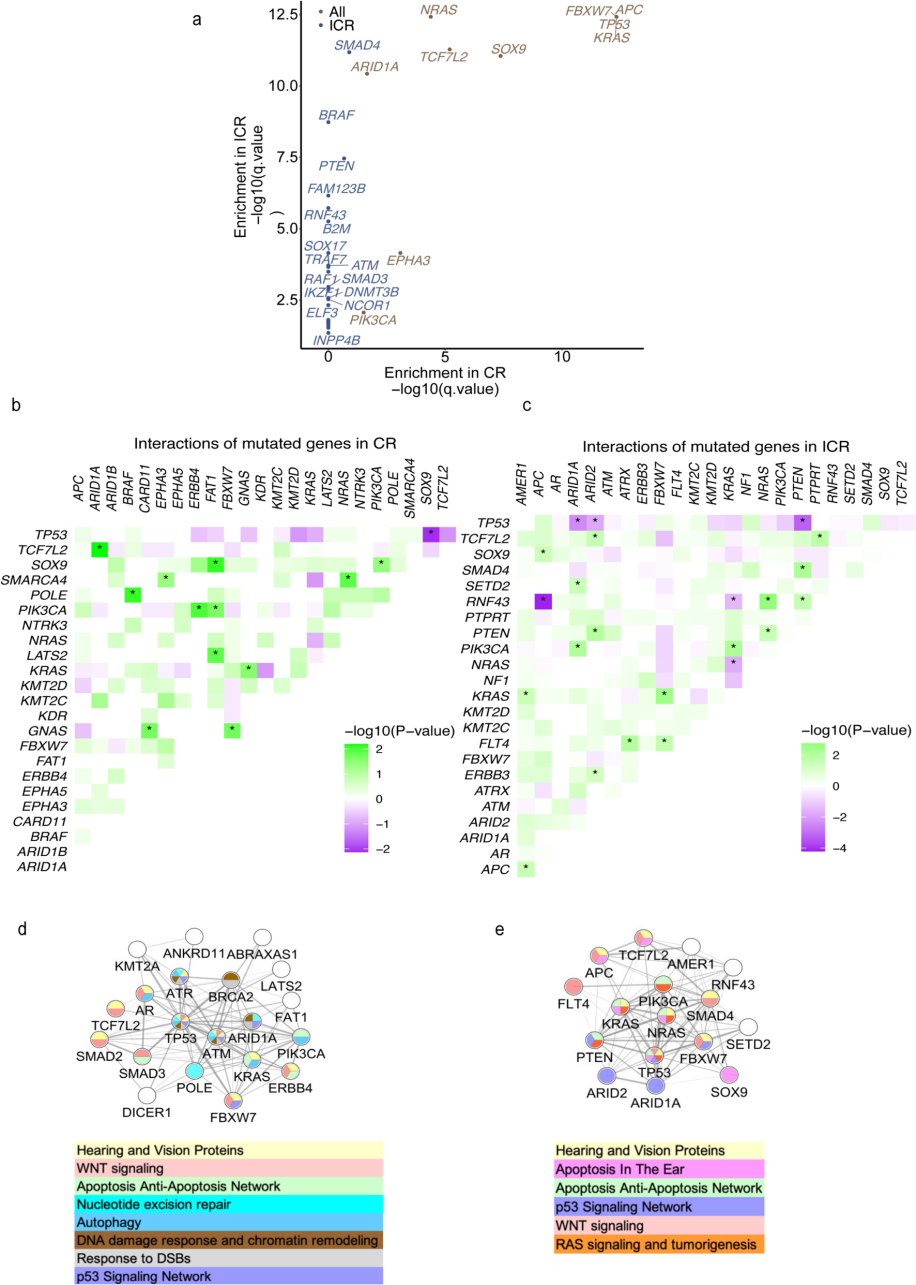
Mutation rates and significantly mutated genes (SMGs) in CR and ICR tumors. **A **Dot plots showing Significantly Mutated Genes (SMGs) enriched in CR tumors (lightsteelblue, *n* = 104), ICR tumors (darkseagreen, *n* = 260), and both groups (wheat), *q*-value < 0.05 after correction for multiple hypothesis testing. **B**-**C **Diagrams depicting interactions between frequently mutated genes in CR tumors (**B**) and ICR tumors (**C**), with green indicating co-occurring mutations and purple showing mutually exclusive mutations. An asterisk in **B** and **C** signifies a significant interaction determined by Fisher's exact test are marked with stars (*p* < 0.05). **D**-**E **Networks of co-occurring mutated genes in CR tumors (**D**) and ICR tumors (**E**)

Next, we used MutSig2CV [27] to identify significantly mutated genes (SMGs) for CR and ICR tumors, respectively. MutSig2CV identifies genes that are mutated more frequently than expected by chance, considering three factors: background mutation rate, localized hotspots, and vertebrate conservation. We discovered 35 SMGs in ICR tumors and 10 in CR tumors (Fig. 2A, Supplementary Table 2). Several well-known cancer driver genes, including *TP53, APC, KRAS, ARID1A, TCF7L2,* and *FBXW7,* were present in both groups. All SMGs in CR tumors were also presented in ICR tumors. However, ICR tumors exhibit more known cancer drivers, such as *SMAD4, BRAF, PTEN, SMAD3, and SOX17*) that were not SMGs in the CR tumors.

In cancer, certain mutations exhibit evolutionary dependencies [46], reflecting interactions between genes. Alterations in one gene may influence the benefit of a particular mutation pattern of another, thereby creating a complex network of mutually exclusive or co-occurring mutation pairs [47]. To explore whether specific genetic interactions are associated with the response to nCRT, we detected significantly co-occurring and mutually exclusive mutation pairs separately within CR and ICR tumor groups. While both CR tumors and ICR tumors demonstrated significantly co-occurring mutations in cancer-related genes(Fisher’s exact test, *p*-value < 0.05), the gene pairs were different between groups with 12 co-occurring pairs in CR tumors compared to 17 pairs in ICR tumors (Fig. 2B and C, Supplementary Data 1,). Additionally, we identified 6 significantly mutually exclusive mutation pairs in ICR tumors and 1 in CR tumors (Fisher’s exact test, *p*-value < 0.05) (Supplementary Data 1). Among these mutually exclusive mutation pairs in ICR tumors, the mutually exclusive alterations in *APC* and *RNF43* has been reported in microsatellite-unstable colorectal adenocarcinomas [48]. Mutations in *PTEN* and *TP53* were also reported exclusive in a subgroup of colorectal cancer [49] and breast cancer [50]. Similar exclusivity between *TP53* and *ARID1A* has been observed in other cancer types [51]. When we compared the network involved in genes with co-occurring or mutually exclusive mutations, in both CR and ICR tumors, those involved in apoptosis or anti-apoptosis network and WNT signaling. Only the CR tumors included autophagy, DNA damage response and chromatin remodeling, response to double strand breaks, and nucleotide excision repair and only the ICR tumors included a network for RAS signaling and tumorigenesis (Fig. 2D and E). CR and ICR tumors exhibit differences in their networks of co-occurring or mutually exclusive genes.

Transcriptomic differences and tumor immune cell infiltrate in response to nCRT.

**Fig. 3:**
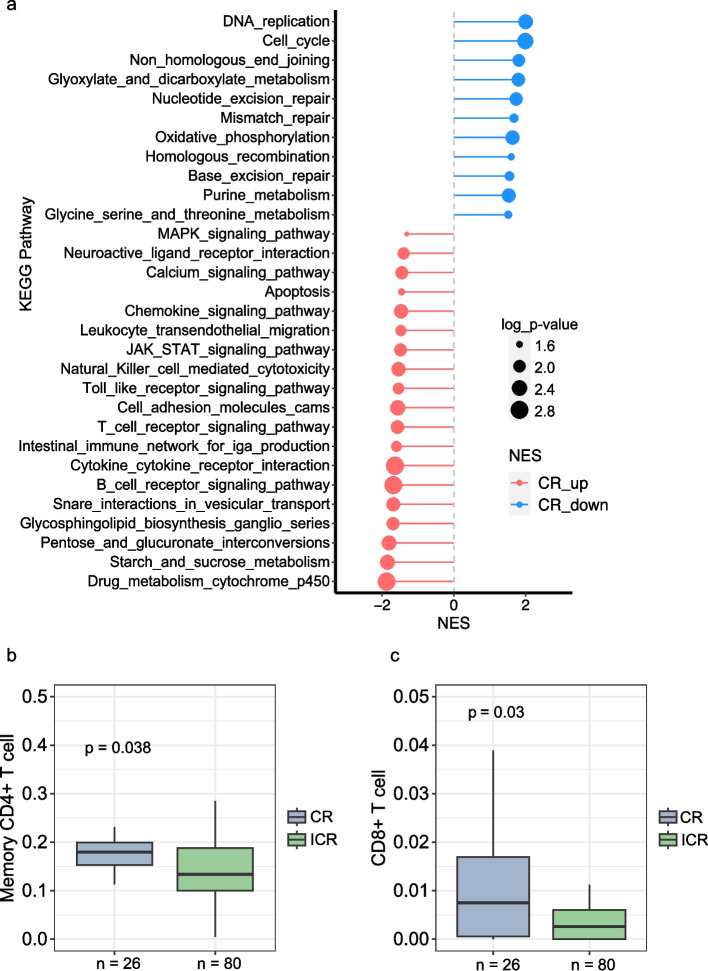
Differences in transcriptomic profile and immune cell infiltration between CR and ICR to nCRT. **A **Pathway Enrichment Analysis. The chart displays significantly enriched KEGG pathways based on differentially expressed genes (DEGs) in complete responders (CR). Pathways more active in CR are indicated in blue (CR down) and those less active in red (CR up). The size of the dot correlates with the -log10 p-value, indicating the statistical significance of the enrichment. **B-C **Box plots comparing the infiltration levels of memory CD4 + T cells (**B**) and CD8 + T cells (**C**) within tumor microenvironments between CR (*n* = 26) and ICR (*n* = 80) groups. Statistical significance was determined using the Mann–Whitney U test, with * indicating *p* < 0.05 and ** indicating *p* < 0.01

To compare the transcriptomic differences between CR and ICR tumors, we merged the RNA sequencing data from 99 patients from the MSK cohort and 7 patients from the UM cohort. The differential expressed genes in ICR tumors were consistent with the study from Chatila *et.al*^*20*^, *IGF2* and *L1CAM* were distinctly overexpressed in ICR tumors. Gene Set Enrichment Analysis (GSEA) revealed that genes with low expression in CR tumors were significantly enriched in several DNA repair-associated pathways, including non-homologous end joining (NHEJ), NER, mismatch repair (MMR), homologous recombination (HR), and Base-excision repair (BER) pathways (Fig. 3A). CR tumors also demonstrated overexpression of numerous genes that were significantly enriched in immune-related pathways (Fig. 3A). Again, of note, MSI-H tumors were excluded from the analysis. Overall this gene expression analysis suggests that decreased DNA repair capability and increased immune function in CR tumors may be important in response to treatment.

Emerging evidence shows that tumor-infiltrating immune cells (TICs) are associated with sensitivity to nCRT [52, 53]. To investigate the differences in TICs between CR and ICR tumors, we employed TIMER [36] to estimate immune infiltrates from the RNA sequencing data and subsequently assessed the differential fractions/enrichment scores. We observed that CR tumors manifested a significantly reduced infiltration of CD8 + T cells and memory CD4 + T cells (Mann Whitney U test, *p*-value < 0.05) (Fig. 3B-C). Although not reaching statistical significance (Mann Whitney U test, *p*-value > 0.05), ICR tumors exhibited lower fractions/enrichment scores across a spectrum of immune cells, including macrophages (M0 and M2), dendritic cells, activate neutral killer (NK) cells, and monocytes (Supplementary Fig. 3, Supplementary Data 2).

Predictors of complete response to neoadjuvant therapy in rectal cancer.

To elucidate both clinical and genomic predictors of complete response (CR), we selected patients with clinical stage 2 and 3 who received neoadjuvant therapy and for whom targeted or whole exome DNA sequencing data was available. This resulted in 226 ICR and 92 CR patients for analysis. Clinical and demographic information for these patients is provided in supplementary Table 1A. To determine what to include in the model bivariate analysis of demographic and clinical variables was performed and p value for inclusion in the model was set at p < 0.02. This bivariate analysis did not show any significant associations with response at p < 0.05 and only age, tumor size, and stage had a p < 0.2. (Supplementary Data 1B). All identified co-occurring mutations found to be significant in CR or ICR patients individually were compared for their presence in CR vs ICR on bivariate analysis and identified a subset of 11 that were more commonly found in CR than ICR patients with p-values < 0.2 (Supplementary Data 3). Next, the selected patient and tumor characteristics consisting of age, tumor size (cm), clinical stage, and number of CR associated co-occurring mutations were included in a logistic regression model for predictors of CR. In the completed model, presence of more of the co-occurring mutations was associated with greater likelihood of CR (OR: 7.4, 95%CI: 3.2—21.0, p < 0.001) after adjusting for age, tumor size, and stage. For each additional co-occurring mutation of the 11 identified to be associated with CR, patients had a 7.4 times higher odds ratio for complete response (Table 1).

**Table 1:** Logistic regression model coefficients for prediction of complete response (CR) to treatment

*Predictors*	*Odds Ratios*	*95% CI*	*p* value
Tumor size (cm)	0.97	0.93–1.00	0.11
AJCC Tumor Stage Classification			
2	Ref		
3	0.63	0.33–1.24	0.17
Number of co-occurring mutations associated with CR at *p* < 0.2	7.41	3.23–21.0	< 0.001
*Observations*	298		
*R*^*2*^* Tjur*	0.135		

*CI *Confidence interval

Genomic and molecular features of incomplete responder tumors that subsequently develop metastasis.

**Fig. 4:**
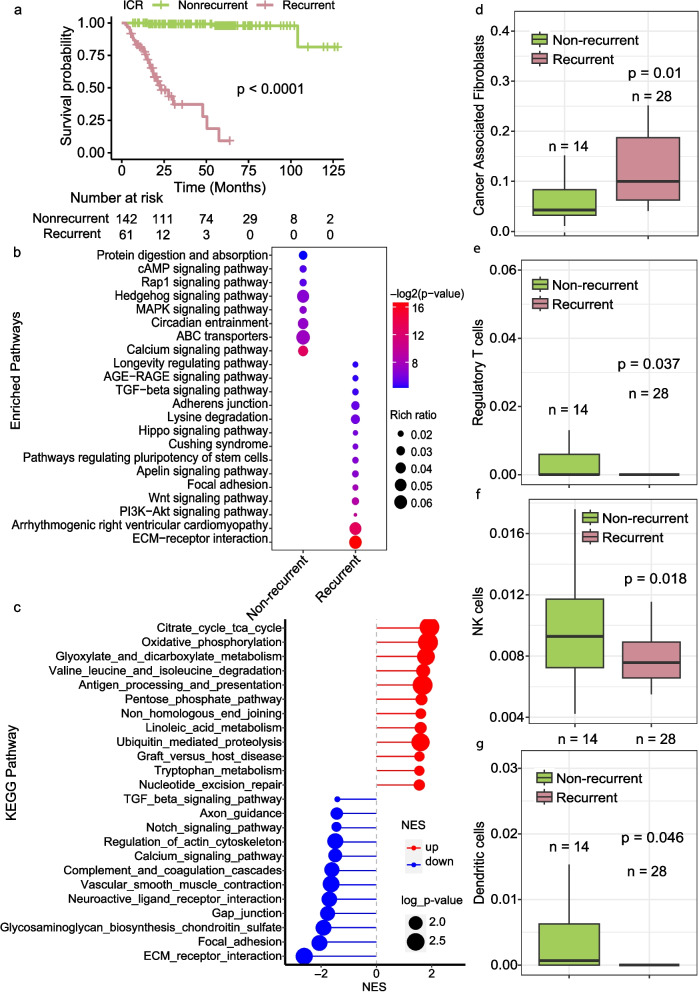
Genomic and immune landscape differences associated with recurrence in incomplete responders to nCRT. **A **Kaplan–Meier Curve showing the survival difference between recurrent (those who developed distant metastasis) (*n* = 56) and non-recurrent (*n* = 153) cases. **B **Enrichment analysis of KEGG pathways comparing frequently mutated genes in recurrent versus non-recurrent ICR tumors, with pathway significance denoted by color intensity and dot size representing the rich ratio. **C **Enrichment plot illustrating the significantly enriched pathways for differentially expressed genes (DEGs) between recurrent and non-recurrent ICR tumors, with upregulated pathways in red and downregulated in blue. **D**-**G **Box plots quantifying the relative abundance of cancer-associated fibroblasts (**D**), regulatory T cells (**E),** NK cells (**F**), and dendritic cells (**G**) in recurrent (*n* = 14) compared to non-recurrent ICR tumors (*n* = 28). Statistical significance determined by Mann–Whitney U test

Given that approximately one-third of ICR tumors subsequently recurred, we explored the molecular features associated with recurrence in these pre-treatment biopsy samples, aiming to enhance our understanding and explore potential strategies for future studies of recurrent ICR tumors. Of the ICR tumors, 6 patients went on to have a local recurrence and 56 developed a distant recurrence (metastasis). We selected out just the tumors where the patients developed distant metastasis and compared them to the ICR, non-recurrent tumors. ICR tumors where the patients developed metastasis had significantly (the log rank test, *p*-value < 0.0001) poorer survival (Fig. 4A). There was no significant difference in their tumor mutation burden, altered genome fractions, or MATH scores [54] (Supplementary Fig. 4A-C). MutSig was used to compare significantly mutated genes between recurrent and non-recurrent ICR tumors with many overlapping genes identified between the groups but only RNF43 being found in the recucrrent ICR tumors (Supplementary Fig. 4D). ICR tumors that later reoccurred exhibited a higher frequency of mutations in genes that are enriched in pathways related to TGF-ß signaling, ECM-receptor interaction, and cell–cell interaction (focal adhesion and adherens junction) (Fig. 4B). In addition, gene expression profiles indicated a downregulation of cell–cell interaction genes in recurrent ICR tumors (Fig. 4C). Moreover, more Cancer Associated Fibroblasts (CAFs), known to influence colorectal cancer (CRC) metastasis [55], were significantly higher in the recurrent ICR tumors (Fig. 4D, p-value = 0.01). Consistent with enriched mutations and decreased expression of cell–cell interaction genes, targeting CAFs may be a potential therapeutic strategy to prevent metastasis in CRC [56, 57]. Additionally, recurrent tumors demonstrated significantly reduced infiltration of NK cells, dendritic cells, and Regulatory T Cells (Tregs) (Fig. 4E-G).

### Characteristics of subgroups of incomplete responders

**Fig. 5:**
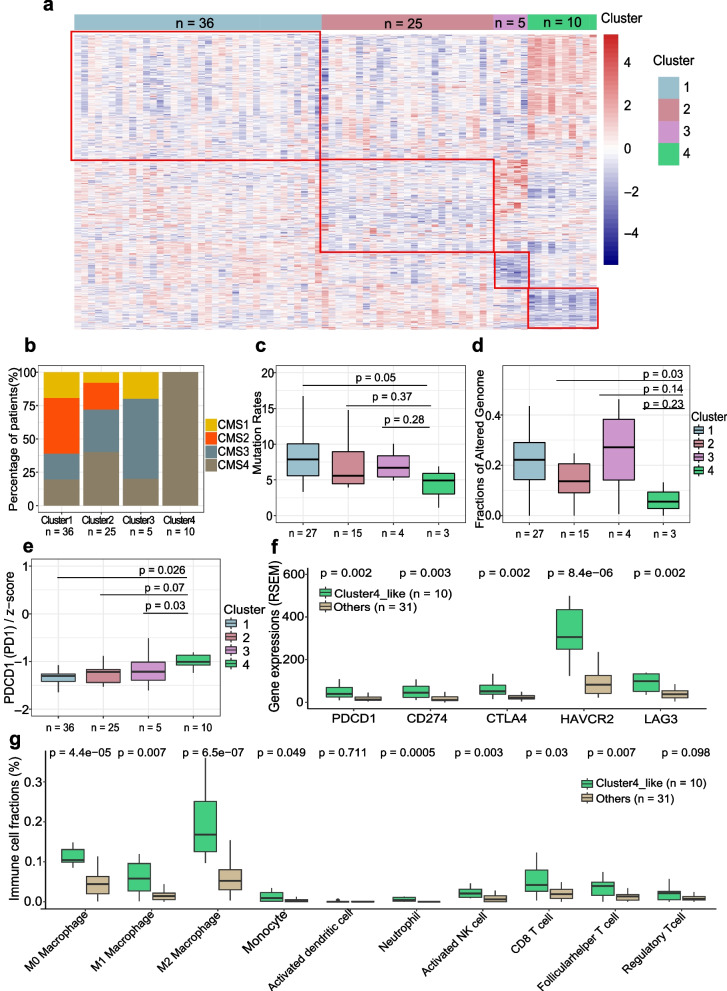
Gene expression profiling reveals an immune-active tumor subtype in incomplete responders associated with increased resistance and poorer prognosis post-neoadjuvant therapy. **A **Heatmap showing distinct gene expression profiles across four identified subgroups of ICR tumors (*n* = 76; Cluster1: *n* = 36, Cluster2: *n* = 25, Cluster3: *n* = 5, Cluster4: *n* = 10). **B **Distribution of Consensus Molecular Subtype (CMS) classifications within each ICR tumor subgroup. **C-D **Boxplots detailing comparison of mutation rate (**C**) and fraction of altered genome (**D**) among the four ICR subgroups (Mann Whitney U test, total *n* = 39, Cluster 1: *n* = 27, Cluster 2: *n* = 15, Cluster 3: *n* = 4, Cluster 4: *n* = 3). **E **Boxplot comparing PD1 gene expression across the four ICR subgroups (Mann Whitney U test, *n* = 76, Cluster 1: *n* = 36, Cluster 2: *n* = 25, Cluster 3: *n* = 5, Cluster 4: *n* = 10). **F **Comparisons of immune checkpoint genes (PD1, PD-L1, CTLA4, HAVCR2, LAG3) expression in the TCGA READ dataset, with a focus on contrasting Cluster 4-like with other clusters (Mann Whitney U test, *n* = 41, Cluster 4-like: *n* = 10, Others: *n* = 31). **G **Assessment of Immune cell fractions within the TCGA READ dataset, showcasing differences between Cluster 4-like and other clusters (Mann Whitney U test, *n* = 41, Cluster 4-like: *n* = 10, Others: *n* = 31)

Given the evidence [58] that gene expression has a higher predictive capacity for drug response than other factors such as mutation signature, copy number variation, and methylation, we aimed to identify ICR patient subgroups based on gene expression patterns. Our objective was to understand the molecular characteristics of these subgroups, potentially guiding alternative therapeutic approaches. Through unsupervised hierarchical clustering of 76 ICR patients with whole exome and transcriptome sequencing and outcome data (Supplementary Data 4), we identified four groups with distinct gene expression profiles (Fig. 5A, Supplementary Fig. 5 A).

Cluster 1 tumors (n = 36) exhibited characteristics similar to CR tumors, marked by elevated mutation rates, enhanced cell–cell interaction, and diminished expression of DNA repair genes (Supplementary Fig. 5B). Cluster 2 tumors (n = 25) showed efficient DNA repair capabilities, albeit with reduced expression of pathways associated with drug metabolism (Supplementary Fig. 5 C). Cluster 3 (n = 5) exhibited a significant upregulation of cytokine and chemokine signaling pathways (Supplementary Fig. 5D), suggesting potential opportunities for cytokine-based therapeutic interventions [59]. Interestingly, all tumors in Cluster 4 (n = 10) were CMS4 subtype (Fig. 5B), which is associated with poorer survival [60, 61]. This tumor subset exhibited a higher proportion of patients with recurrence (25% in Cluster 1 compared to 40% in Cluster 4, Supplementary Fig. 5E). Additionally, these tumors displayed distinct genomic alternations, including decreased mutation rates and lower fraction of altered genomes (Fig. 5C-D), along with lower expression of several cell–cell interaction pathways (Supplementary Fig. 5 F). Among the top 50 DEGs between tumors in Cluster 4 and other clusters, we identified 4 genes (*DUOXA2*, *DUOX2*, *SSH1*, *FRMD6*) that were significantly associated with worse survival (Supplementary Fig. 5G-J). Previous studies have reported that *DUOX2* and *SSH1* exhibited significantly higher expression and promoted the progression and metastasis of CRC [62, 63]. Additionally, *FRMD6*, which is an upstream regulator of Hippo signaling, serves as a marker of the CMS4 subgroup in CRC [64, 65].

Furthermore, the immune microenvironment of Cluster 4 tumors was characterized by increased infiltration of various immune cells, including neutrophils, monocytes, macrophages, activated myeloid dendritic cells, naive CD4 + T cell, as well as Tregs cells (Supplementary Fig. 5 K-P). The immune-rich microenvironment of Cluster 4 may indicate that there is an opportunity for immunotherapy for this specific subset of tumors. Of note, we observed significant overexpression of *PDCD1* (PD-1) in Cluster 4 (Fig. 5E), highlighting its potential as a therapeutic target [66, 67].

To validate these findings, we applied the differential expression signature between Cluster 4 and other clusters to 42 rectum adenocarcinoma (READ) samples in TCGA. We identified that a subgroup, termed Cluster4-like, demonstrated a similar expression profile to Cluster 4 (Supplementary Fig. 5Q-R). Interestingly, the tumors in the Cluster4-like group (n = 10) also had poor outcomes, with 80% being CMS4 subtype (Supplementary Fig. 5S). Immune checkpoint genes, including PD-1, *CD274* (PD-L1), *CTLA4*, *HAVCR2* (TIM3), and *LAG3,* were overexpressed in Cluster4-like tumors (Fig. 5F). These tumors also displayed increased immune cell infiltration (Fig. 5G). These differences mirrored Cluster 4 characteristics. Collectively, our findings suggest that attention should be given to potentially identifying subsets of patients with high immune cell infiltration and overexpression of immune checkpoint genes, as these patients might be candidates to study immune checkpoint inhibitors (ICIs). These data reinforce the findings of other studies that have shown there are subsets of patients with immune-rich tumors.

## Discussion

Rectal cancer treatment is evolving. Recent clinical studies have focused on optimizing treatment by altering the order of chemotherapy and chemoradiotherapy relative to surgery and have identified patients where organ preservation appears feasible such that patients can avoid surgical morbidity if they appear to have a clinical complete response and instead be followed with a watch and wait approach. Yet, molecular predictors of treatment response and mechanisms of resistance remain unidentified [5]. In this current study, we have gathered public and institutional genomic data from pre-treatment biopsies of patients undergoing chemoradiotherapy, followed by clinical response assessment or surgery. Our in-depth comparative analysis sought to answer the 2 most fundamental clinical questions in the care of rectal cancer patients: how CR and ICR patient tumors differ prior to treatment and how tumors from patients who later will recur differ from those who don’t. We identified multiple important differences, some of which are potentially targetable. We utilized advanced bioinformatics approaches for these comparisons, including the use of the propensity score weighting to balance confounding factors that may have been associated with genetic changes, such as BMI, age, gender, tumor size, and N and T stages.

Our comprehensive analysis has identified important molecular and clinical factors influencing the response to neoadjuvant chemoradiotherapy (nCRT). We identified that ICR patients presented with larger tumor sizes and go on to have higher recurrence rates compared to CR patients. Both CR and ICR patients have particular (but differing) co-occurring and mutually exclusive DNA mutations. CR tumors were enriched in DNA repair alterations and DNA damage response networks. Transcriptomic analysis revealed that ICR tumors had increased expression of DNA repair genes, while CR tumors had increases in genes in immune pathways. The immune microenvironment of CR tumors had significantly higher infiltrating memory CD4 + T cells and CD8 + T cells. Using logistic regression analysis, we identified particular co-occurring mutations as potential CR predictors for future studies. Taking into account tumor size and AJCC tumor stage, for each of these co-occurring mutations identified, the odds of a patient being a CR are 7.41 fold greater. Our study also revealed unique characteristics for ICR tumors that later recurred, including increased TGF-ß signaling and impaired cell–cell interaction. Lastly, we identified four distinct ICR subgroups based on gene expression, each suggesting different therapeutic strategies. Specifically, the Cluster-4 group, with a CMS-like tumor expression profile, demonstrated the greatest resistance to nCRT and most immune infiltration, suggesting the potential for targeted checkpoint inhibitor therapy to improve the outcome in this challenging patient subgroup. These findings will require validation in a separate cohort since this validation was not performed as part of our study. The only prior study showing response to immune checkpoint therapy was in MSI-high tumors and showed a remarkable complete response in the small number of patients studied [68]. MSI patients were not included in our analysis due to their their rarity and the differences in the genetic mechanisms causing these tumors. No study targeting the patients identified in our study with immune checkpoint therapy has been performed.

Rectal cancer is a complex and molecularly heterogeneous disease [69]. For ICR tumors, an additional objective was to identify subsets that may be responsive to alternative treatments. We did not find that the particular treatment regimen used was associated with CR or ICR and prior studies have been mixed with some showing that incomplete response is not different between TNT and 5FU chemoradiotherapy alone [8]. We first investigated the molecular features of recurrent ICR tumors. Compared to nonrecurrent ICR tumors, recurrent counterparts had no significant differences in TMB or genome alterations (Supplementary Fig. 4A-B). However, ICR tumors which later recurred, displayed a higher frequency of mutations in cell–cell interaction pathways, including focal adhesion, adherens junction, TGF-ß signaling, and ECM-receptor interaction. Moreover, the expression data found diminished activity in these pathways, suggesting impaired cell–cell interaction is characteristic of these tumors. Therefore, strategies targeting this dysregulation may benefit patients with these alterations which we identified in ICR tumors that went on to recur. Interestingly, these differences are already identifiable in pre-treatment biopsies indicating that tumors harbor alterations which cause subsequent metastasis at diagnosis.

Subsequently, we identified a subset of ICR tumors using gene expression profiles and validated these using TCGA data. This patient subset, characterized by poor outcomes, exhibited a reduced mutation burden and genomic alterations but displayed significantly increased immune cell infiltration. Moreover, the over-expression of immune checkpoint mRNAs suggests a potential responsiveness to immune checkpoint inhibitors.

While our study has identified multiple potential predictors of response and recurrence, it also has important limitations. We have collected publicly available data and our own institutional data from patients treated in different ways as part of multiple trials. However, all of the patients included had stage 2 or 3 rectal cancer, had MSS tumors, and received long-course chemoradiation. Because we included patients from various setting, our findings are likely more applicable. Additional limitations include sample size but to our knowledge, this is the largest study of this kind to date. Another limitation includes the lack of validation via immunohistochemistry. Most of the samples were part of a national study so we did not have access to the samples for this sort of validation. An additional limitation was the lack of use of a secondary cohort to validate our regression analysis.

In conclusion, we assessed for molecular differences in pre-treatment biopsies of tumors where patients went on to be CRs or ICRs. We identified several molecular differences in these tumors and developed a model to predict response which will need to be validated in a separate cohort. In addition, we assessed and identified molecular characteristics of ICR tumors where the patients develop a later metastasis. Lastly, we used unsupervised hierarchical clustering to identify subsets of ICR tumors and identified similar findings in TCGA tumors. This analysis utilized our own data with publicly available data to add to our understanding of the complexity of rectal cancer.

## Supplementary Information

Supplementary Material 1.

Supplementary Material 2.

## Data Availability

All data are available from the corresponding authors upon reasonable request. For RNA-seq data has been deposited in the Gene Expression Omnibus database under accession GSE242786, and the public dataset under accession GSE209746.
