# Supplementary material for: Molecular predictors of treatment resistance and recurrence following neoadjuvant therapy in rectal cancer
Source: BMC Cancer. 2025 Oct 22;25:1624. doi: 10.1186/s12885-025-14958-4 (PMC12542088; doi:10.1186/s12885-025-14958-4)
Supplement: Supplementary file 2 — Supplementary Material 2. [file 12885_2025_14958_MOESM2_ESM.docx]

**Supplementary Table 1.** Bivariate of response and clinical factors.

|  | ICR (N=226) | CR (N=92) | Total (N=318) | *p* value |
| --- | --- | --- | --- | --- |
| Cohort |  |  |  | 0.45 (1) |
| MSK, Timing | 210 (92.9%) | 88 (95.7%) | 314 (93.7%) |  |
| UM | 16 (7.1%) | 4 (4.3%) | 20 (6.3%) |  |
| Sex |  |  |  | 0.53 (1) |
| Female | 90 (40.5%) | 41 (45.1%) | 131(41.9%) |  |
| Male | 132 (59.5%) | 50 (54.9%) | 182 (58.1%) |  |
| Age |  |  |  | 0.08 (2) |
| Mean (SD) | 53.9 (13.1) | 56.7 (12.7) | 54.7 (13.0) | |
| Tumor size (cm) |  |  |  | 0.09 (3) |
| Median (Q1, Q3) | 4.6 (3.8, 6.0) | 4.2 (3.3, 5.3) | 4.5 (3.6, 5.8) | |
| Distance from av (cm) | |  |  | 0.91 (2) |
| Mean (SD) | 6.6 (3.1) | 6.7 (3.1) | 6.6 (3.1) |  |
| Rectal location |  |  |  | 0.57 (1) |
| Lower | 47 (22.4%) | 20 (23.5%) | 67 (22.7%) |  |
| Middle | 95 (45.2%) | 33 (38.8%) | 134 (43.4%) |  |
| Upper | 68 (32.4%) | 32 (37.6%) | 100 (33.9%) |  |
| AJCC classification | |  |  | 0.12 (1) |
| 2 | 38 (16.8%) | 23 (25.0%) | 61 (19.2%) |  |
| 3 | 188 (83.2%) | 69 (75.0%) | 257 (80.8%) |  |
| T stage |  |  |  | 0.25 (1) |
| 2 | 5 (2.9%) | 6 (9.2%) | 11 (4.7%) |  |
| 3 | 144 (84.2%) | 51 (78.5%) | 195 (82.6%) |  |
| 4 | 22 (12.9%) | 8 (12.3%) | 30 (12.7%) |  |
| Ln positive |  |  |  | 0.18 (1) |
| No | 25 (14.1%) | 14 (21.2%) | 39 (16.0%) |  |
| Yes | 152 (85.9%) | 52 (78.8%) | 204 (84.%) |  |
| Race |  |  |  | 0.23 (1) |
| Asian | 15 (7.1%) | 7 (8.0%) | 22 (7.4%) |  |
| Black | 8 (3.8%) | 4 (4.5%) | 12 (4.0%) |  |
| Others | 37 (17.6%) | 24 (27.3%) | 61 (20.5%) |  |
| White | 150 (71.4%) | 53 (60.2%) | 203 (68.1%) |  |

*p* values calculated by 1. Fisher’s exact test, 2. Student’s t-test, 3. Wilcoxon rank sum test

**Supplementary Table 2.** Significantly mutated genes (SMGs) detected by MutSig2CV in CR and ICR groups. Ranked by q-value

| SMGs | q-value | Group |
| --- | --- | --- |
| APC | 3.77E-13 | ICR |
| TP53 | 3.77E-13 | ICR |
| KRAS | 3.77E-13 | ICR |
| FBXW7 | 3.77E-13 | ICR |
| NRAS | 3.77E-13 | ICR |
| TP53 | 4.72E-13 | CR |
| APC | 4.72E-13 | CR |
| KRAS | 4.72E-13 | CR |
| FBXW7 | 4.72E-13 | CR |
| TCF7L2 | 5.24E-12 | ICR |
| SMAD4 | 6.58E-12 | ICR |
| SOX9 | 8.90E-12 | ICR |
| ARID1A | 3.72E-11 | ICR |
| BRAF | 1.85E-09 | ICR |
| PTEN | 3.46E-08 | ICR |
| SOX9 | 4.28E-08 | CR |
| FAM123B | 6.93E-07 | ICR |
| RNF43 | 1.89E-06 | ICR |
| B2M | 5.53E-06 | ICR |
| TCF7L2 | 6.45E-06 | CR |
| NRAS | 4.16E-05 | CR |
| EPHA3 | 7.09E-05 | ICR |
| SOX17 | 7.09E-05 | ICR |
| ATM | 1.95E-04 | ICR |
| TRAF7 | 2.19E-04 | ICR |
| RAF1 | 3.21E-04 | ICR |
| EPHA3 | 8.29E-04 | CR |
| SMAD3 | 1.09E-03 | ICR |
| IKZF1 | 1.34E-03 | ICR |
| DNMT3B | 2.67E-03 | ICR |
| NCOR1 | 2.96E-03 | ICR |
| ELF3 | 4.76E-03 | ICR |
| PIK3CA | 8.64E-03 | ICR |
| KDM6A | 1.57E-02 | ICR |
| ARID2 | 1.72E-02 | ICR |
| AXIN2 | 1.79E-02 | ICR |
| HGF | 1.96E-02 | ICR |
| ERBB3 | 2.08E-02 | ICR |
| NTRK1 | 2.10E-02 | ICR |
| MAPK1 | 2.16E-02 | ICR |
| ARID1A | 2.21E-02 | CR |
| CTCF | 2.47E-02 | ICR |
| ASXL2 | 2.96E-02 | ICR |
| PIK3CA | 3.09E-02 | CR |
| INPP4B | 4.44E-02 | ICR |

q-value is FDR-corrected (Benjamini-Hochberg) overall p-value

**Supplementary Table 3.** Significant mutated genes associated with response.

|  | ICR (N=226) | CR (N=92) | Total (N=318) | p value |
| --- | --- | --- | --- | --- |
| ***AMER1*** |  |  |  | 0.294 (1) |
| mutation not present | 211 (93.4%) | 89 (96.7%) | 300 (94.3%) |  |
| mutation present | 15 (6.6%) | 3 (3.3%) | 18 (5.7%) |  |
| ***ATM*** |  |  |  | 0.206 (1) |
| mutation not present | 209 (92.5%) | 89 (96.7%) | 298 (93.7%) |  |
| mutation present | 17 (7.5%) | 3 (3.3%) | 20 (6.3%) |  |
| ***CTCF*** |  |  |  | 0.187 (1) |
| mutation not present | 220 (97.3%) | 92 (100.0%) | 312 (98.1%) |  |
| mutation present | 6 (2.7%) | 0 (0.0%) | 6 (1.9%) |  |
| ***NCOR1*** |  |  |  | 0.199 (1) |
| mutation not present | 219 (96.9%) | 92 (100.0%) | 311 (97.8%) |  |
| mutation present | 7 (3.1%) | 0 (0.0%) | 7 (2.2%) |  |
| ***SMAD4*** |  |  |  | 0.120 (1) |
| mutation not present | 203 (89.8%) | 88 (95.7%) | 291 (91.5%) |  |
| mutation present | 23 (10.2%) | 4 (4.3%) | 27 (8.5%) |  |
| ***SOX17*** |  |  |  | 0.291 (1) |
| mutation not present | 217 (96.0%) | 91 (98.9%) | 308 (96.9%) |  |
| mutation present | 9 (4.0%) | 1 (1.1%) | 10 (3.1%) |  |
| ***ARID1B*** |  |  |  | 0.210 (1) |
| mutation not present | 219 (96.9%) | 86 (93.5%) | 305 (95.9%) |  |
| mutation present | 7 (3.1%) | 6 (6.5%) | 13 (4.1%) |  |
| ***EPHA3*** |  |  |  | 0.276 (1) |
| mutation not present | 216 (95.6%) | 85 (92.4%) | 301 (94.7%) |  |
| mutation present | 10 (4.4%) | 7 (7.6%) | 17 (5.3%) |  |
| ***TCF7L2*** |  |  |  | 0.120 (1) |
| mutation not present | 211 (93.4%) | 81 (88.0%) | 292 (91.8%) |  |
| mutation present | 15 (6.6%) | 11 (12.0%) | 26 (8.2%) |  |

*p* value was calculated using Fisher’s Exact Test

**Supplementary Figure 1. Cohort overview and Comparison of clinical factors between complete responders and incomplete responders. A: Overview of the analysis and datasets. B:** Statistical comparison of clinical variables (left) and clinical outcomes (right) between CR (n=92) and ICR tumors (n=226).The continuous factors: age, BMI, tumor size, distance from AV, overall survival (OS), disease-free survival (DFS) from neoadjuvant therapy (NEO), DFS from surgery, were compared using Mann Whitney U test. The discrete factors: sex, race, clinical T stage, N stage, M stage, AJCC classification, OS status, DFS from NEO status, and recurrence, were compared using Chi-squared test. Factors with p < 0.05 are highlighted in red, indicating a statistically significant difference between CR and ICR groups, while those with p > 0.05 are in gray, indicating no significant difference.

**Supplementary Figure 2. Propensity Score Weighting (PSW) analysis for mutation burden and the mutation frequencies in CR and ICR tumors. A:** The forest plot displays standardized mean differences of confounding factors between the groups before (Unadjusted) and after applying PSW. **B:** Box plot comparing mutation burden (weighted by propensity score) between CR and ICR (Weighted t-test, p=0.46, n=318, with CR:92, ICR:226). **C:** Bar plot showing all mutated genes have higher mutation frequencies in CR (n=92) than ICR tumors (n=226). **D:** Bar plot showing mutated genes which have higher mutation frequencies in ICR (n=226) than CR tumors (n=92).

**Supplementary Figure 3. Differences in transcriptomic profile between CR and ICR to nCRT and Network analysis of predictive mutated genes for treatment response**. Box plots comparing the infiltration levels of immune cells within tumor microenvironments between CR (n=26) and ICR (n=80) groups.

**
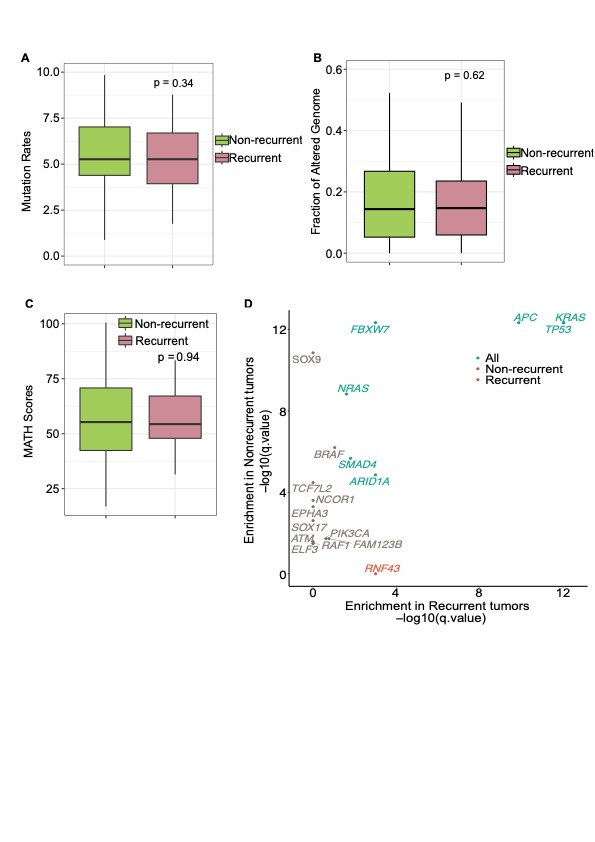
**

**Supplementary Figure 4. Genomic differences and significant mutated genes associated with recurrence in incomplete responders to nCRT. A-C:** Box plots showing the comparison of genomic metrics between recurrent (n=62) and nonrecurrent ICR tumors (n153), including mutation rate **(A),** fraction of altered genome **(B),** and MATH scores **(C)**. No statistically significant differences were observed (Mann Whitney U test, p > 0.05). **D**: Dot plots showing Significantly Mutated Genes (SMGs) enriched in recurrent ICR tumors (red, n=62), non-recurrent ICR tumors (wheat, n=153), and both groups (light green), *q*-value < 0.05 after correction for multiple hypothesis testing.

**Supplementary Figure 5. Additional gene expression profiles in ICR tumor subsets. A**: Distribution of patient samples across four identified subgroups. **B-D, F**: Pathway enrichment analysis for DEGs between specific subgroups (cluster1: **C**, cluster2: **D**, cluster3: **E**, and cluster4: **F**) and all other subgroups. **E**: Fraction of recurrent cases in each ICR patient subgroup. **G-J**: Kaplan-Meier Curve showing the survival difference between tumors with high (> median) and low (< median) expression of DUOXA2 (**G,** n=107, High:48, Low:59), DUOX2 (**H**, n=107, High:50, Low:57), SSH1 (**I**, n=107, High:48, Low:59), and FRMD6 (**J**, n=107, High:61, Low:46) genes (The Log-rank test, p < 0.05). **K-P**: Box plots illustrating the distribution of immune cells (neutrophils (**K**), monocytes (**L**), macrophages (**M**), naïve T CD4 T cell (**N**), dendritic cells (**O**), and regulatory T cells (**P**)) across the four ICR patient subgroups (ANOVA, p < 0.05, n=76, Cluster 1:36, Cluster 2:25, Cluster 3:5, Cluster 4:10). **Q**: Heatmap of the top 50 DEGs between tumors in Cluster 4 (n=10) and other clusters (n=66). **R**: Heatmap of the top 50 DEGs between tumors in Cluster 4-like (n=10) and other clusters (n=31) from the TCGA READ dataset. **S**: Composition of consensus molecular subtypes (CMS) in Cluster4-like and other clusters.
